# Supplementary material for: Escherichia coli metabolism under short-term repetitive substrate dynamics: adaptation and trade-offs
Source: Microb Cell Fact. 2020 May 29;19:116. doi: 10.1186/s12934-020-01379-0 (PMC7260802; doi:10.1186/s12934-020-01379-0)
Supplement: Supplementary file 1 — Additional file 1. Additional file supporting the results discussed in the text. [file 12934_2020_1379_MOESM1_ESM.docx]

**Additional file**

***Escherichia coli* metabolism under short-term repetitive substrate dynamics: Adaptation and trade-offs**

Eleni Vasilakou^1*^, Mark C. M. van Loosdrecht^1^_,_ S. Aljoscha Wahl^1*^

^1^ Department of Biotechnology, Delft University of Technology, Van der Maasweg, 2629 HZ Delft, The Netherlands

1. Repetitiveness of block-wise feeding cycles


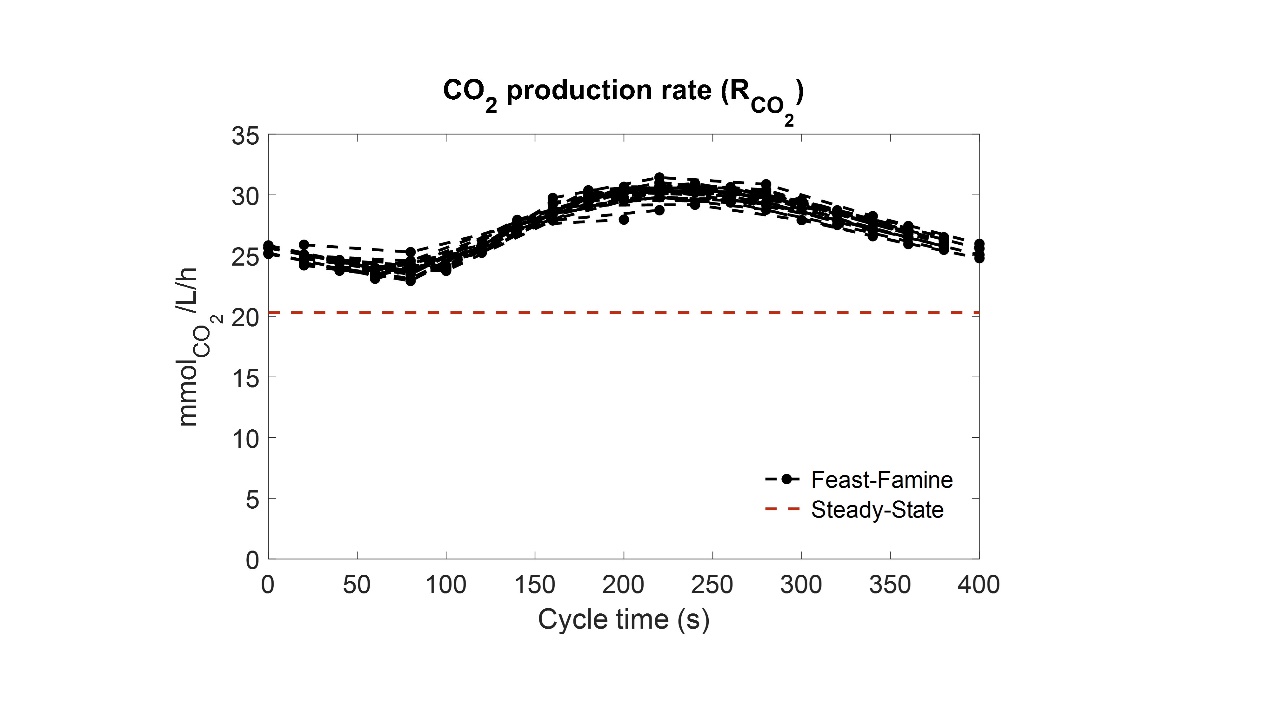


Figure S1 In black: Calculated CO_2_ production rate (mmol_CO2_∙L^-1^∙h^-1^) based on the raw offgas CO_2_ data, over the feast-famine cycle time (s). Data of 30 successive cycles, after at least 8 residence times, are overlapped. These rates are not corrected for delays expected due to headspace, tubing and bicarbonate in the broth. In red: CO_2_ production rate (non-reconciled) during the reference steady-state.

1. Dissolved oxygen

The dissolved oxygen sensor used in this work was a polarographic ADI probe (Applisens, Applikon, Delft, The Netherlands) submerged into the broth. At a polarographic electrode, oxygen is reduced to water (cathode) and the electrons produced generate current, which transmits the signal. These types of probes are known to show some response delays, as a result of many factors, such as the membrane thickness etc. [1]. Because of the short-term behaviour of our experiment (seconds), the time delay of the probe should be taken into account, in order to estimate the real respiration rates. We will describe the oxygen probe dynamics with the following first order model [2]:

|  | $\frac{{dC}_{O2,L}}{dt}= \frac{\left( \hat{C_{O_{2},L}}-C_{O_{2},L} \right)}{\tau_{probe}}$ |  |
| --- | --- | --- |

where $C_{O_{2},L}$ is the dissolved oxygen measured by the sensor (%), $\hat{C_{O_{2},L}}$ is the estimated real dissolved oxygen in the broth (%), *t* is the cycle time (s) and *τ_probe_* is the time (s) needed for the sensor to reach 63.7 % of the ultimate response in a step exchange experiment [3]. The τ_probe_ of our sensor was measured to be 16.65 s. Therefore, the estimated dissolved oxygen in the broth during the feast-famine regime was calculated as follows:

|  | $\hat{C_{O_{2},L}}=C_{O_{2},L}+\tau_{probe}\frac{{dC}_{O_{2},L}}{dt}$ |  |
| --- | --- | --- |

1. Calculation of O_2_ uptake and CO_2_ production rates

In order to calculate the O_2_ uptake and CO_2_ production rates over one cycle time, the rates were first estimated by applying the respective mass balances over time.

The offgas in our system consisted of oxygen, carbon dioxide and nitrogen. Nitrogen gas was not produced or consumed during the cultivation. Therefore the sum of fractions of gases entering and exiting the reactor was 1:

|  | $y_{N_{2},G,in}+y_{O_{2},G,in}+y_{{CO}_{2},G,in}=1$ | |  | |
| --- | --- | --- | --- | --- |
|  | |  | |  |
|  | $y_{N_{2},G,out}+y_{O_{2},G,out}+y_{{CO}_{2},G,out}=1$ | |  | |

where $y_{x,G,in}$ and $y_{x,G,out}$ are the fractions of the respective x gases (N_2_, O_2_ and CO_2_) entering and exiting the reactor, respectively. The fractions of O_2_ and CO_2_ were measured by the offgas analyzer every minute and values for every second were obtained with interpolation.

Applying the nitrogen gas balance:

|  | $F_{G,in}\cdot y_{N_{2},G,in}=F_{G,out}\cdot y_{N_{2},G,out}$ |  |
| --- | --- | --- |

where $F_{G,out}$ and $F_{G,in}$ are the flow rates (mmol_air_ h^-1^) of air exiting and entering the reactor, respectively. In our experimental setup air was provided with a flow rate of 1.875 mmol_air_ h^-1^.

From , and , the gas outflow leaving the reactor was calculated, every second of the cycle:

|  | $F_{G,out}=\frac{F_{G,in}\cdot\left( 1-y_{O_{2},G,in}-y_{{CO}_{2},G,in} \right)}{1-y_{O_{2},G,out}-y_{{CO}_{2},G,out}}$ |  |
| --- | --- | --- |

From the mass balances of O_2_ and CO_2_, the rates of consumption and production were then estimated respectively:

|  | $R_{O_{2}}=F_{G,out}\cdot y_{O_{2},G,out}-F_{G,in}\cdot y_{O_{2},G,in}$ |  |
| --- | --- | --- |
|  | $R_{{CO}_{2}}=F_{G,out}\cdot y_{{CO}_{2},G,out}-F_{G,in}\cdot y_{{CO}_{2},G,in}$ |  |

where $R_{O_{2}}$ (mmol_O2_ h^-1^) and $R_{{CO}_{2}}$ (mmol_CO2_ h^-1^) are the O_2_ consumption and CO_2_ production rates, respectively, for every timepoint in the feast-famine cycle.

The biomass specific rates were then calculated:

|  | $q_{O_{2}}=\frac{R_{O_{2}}}{C_{BM}\cdot V}$, $q_{{CO}_{2}}=\frac{R_{{CO}_{2}}}{C_{BM}\cdot V}$ |  |
| --- | --- | --- |

where C_BM_ is the biomass concentration in the broth (g_CDW_ L^-1^) and V is the broth volume (L).

We performed the above calculations for 16 successive feast-famine cycles and then used the average of all cycles for every second of the cycle.

We then added a pure time delay in both rates, which was assumed to be 46 seconds for O_2_ and 72 seconds for CO_2_, based on the time it took for the offgas O_2_ concentration to decrease and CO_2_ concentration to increase (offgas analyzer) after the beginning of the feeding.

For both rates, a piecewise affine (PWA) rate approximation [4] was calculated. The breakpoints used were timepoints of 0, 20, 50, 80, 135, 262 and 400 s. These breakpoints were chosen, as they exhibited the highest goodness of fit (R^2^ was used), among various combinations [5]. The rates between the breakpoints followed a first order linear function.

Using the measured $y_{O_{2},G,out}$ and $y_{{CO}_{2},G,out}$ ratios and the calculated $q_{O_{2}}$ and $q_{CO_{2}}$ rates, an optimization was performed (Matlab R2018a, The MathWorks, Inc.) by minimizing the sum of squares between the initial measurements and the predicted.

The following differential equations were used for the optimization:

For oxygen:

|  | $\frac{{d\left[ O_{2} \right]}_{out}}{dt}=F_{G,in}\cdot\left[ O_{2} \right]_{in}-F_{G,out}\cdot\left[ O_{2} \right]_{out}-R_{O_{2}}$ |  |
| --- | --- | --- |

where [O_2_] is the concentration of oxygen in the gas phase.

For carbon dioxide:

At pH 7.0 there is significant interconversion of dissolved CO_2_ and bicarbonate in the broth [6], which was taken into account in our model. Using the system described in [7], the following differential equations for CO_2_ and HCO_3_^-^ were derived:

|  | $\frac{{d\left[ {CO}_{2} \right]}_{out}}{dt}=F_{G,in}\cdot\left[ CO_{2} \right]_{in}-F_{G,out}\cdot\left[ {CO}_{2} \right]_{out}+R_{{CO}_{2}}-\left( k_{1}+k_{2}\cdot{10}^{pH-14} \right)\cdot\left[ {CO}_{2} \right]_{out}+\left( k_{-2}+k_{-1}\cdot{10}^{-pH} \right)\cdot\left[ {HCO}_{3}^{-} \right]$ |  |
| --- | --- | --- |
|  | $\frac{d\left[ {HCO}_{3}^{-} \right]}{dt}=\left( k_{1}+k_{2}\cdot{10}^{pH-14} \right)\cdot\left[ {CO}_{2} \right]_{out}-\left( k_{-2}+k_{-1}\cdot{10}^{-pH} \right)\cdot\left[ {HCO}_{3}^{-} \right]$ |  |

where [CO_2_] is the concentration of CO_2_ in the gas phase and [HCO_3_^-^] is the concentration of bicarbonate in the broth. k_1_, k_-1_, k_2_ and k_-2_ are the reaction constants, as described in [7]. For our calculations we used values from the literature for 37^0^C, as follows:

$k_{-1}=60$ in s^-1^ [6]

$k_{1}=e^{-11.582-\frac{918.9}{T}}\cdot k_{-1}$ in M^-1^s^-1^ [8], where T = 310.15 K

$k_{-2}=107\cdot{10}^{-5}$ in s^-1^ [6]

$k_{2}=\frac{e^{-11.582-\frac{918.9}{T}}}{k_{wf}}\cdot k_{-2}$ in M^-1^s^-1^ , where $k_{wf}=e^{148.9802-\frac{13847.26}{T}-23.6521\cdot\ln T}$ is the water dissociation equilibrium constant [9].

1. Extracellular by-products

Table S1 Extracellular by-product concentration measurements in mM for steady-state (3 replicates) and feast-famine (over time).

|  | **Lactate** | **Formate** | **Acetate** | **Ethanol** |
| --- | --- | --- | --- | --- |
| **Steady-state** | | | | |
| **Sample 1** | 4.42 | 0.33 | 1.11 | 9.27 |
| **Sample 2** | 4.53 | 0.33 | 1.12 | 8.62 |
| **Sample 3** | 4.97 | 0.37 | 1.17 | 7.90 |
|  |  |  |  |  |
| **Feast-famine** | | | | |
| **Timepoints (s)** |  |  |  |  |
| **0** | 1.52 | 1.66 | 2.13 | - |
| **2.5** | 1.56 | 1.61 | 1.99 | 0.59 |
| **5** | 1.54 | 1.62 | 2.01 | 2.15 |
| **7.5** | 1.56 | 1.64 | 2.00 | 1.38 |
| **10** | 1.57 | 1.52 | 2.21 | 1.13 |
| **12.5** | 1.60 | 1.76 | 2.47 | 1.85 |
| **15** | 1.54 | 1.75 | 2.39 | 6.58 |
| **17.5** | 1.75 | 1.89 | 2.45 | 3.82 |
| **20** | 1.80 | 1.94 | 2.44 | 4.21 |
| **25** | 1.93 | 2.07 | 2.40 | 3.27 |
| **30** | 1.50 | 1.74 | 2.11 | 4.99 |
| **40** | 1.51 | 1.74 | 2.45 | 3.34 |
| **50** | 1.67 | 1.77 | 2.33 | 4.74 |
| **60** | 1.66 | 1.89 | 2.46 | 2.64 |
| **70** | 1.64 | 1.87 | 2.55 | 2.60 |
| **80** | 1.81 | 1.95 | 2.37 | 2.98 |
| **90** | 1.56 | 1.63 | 2.07 | 2.52 |
| **95** | 1.64 | 1.88 | 2.40 | 4.89 |
| **100** | 1.54 | 1.63 | 2.06 | - |
| **110** | 1.50 | 1.74 | 1.93 | 3.91 |
| **120** | 1.96 | 2.14 | 2.46 | 3.73 |
| **135** | 1.56 | 1.66 | 2.13 | - |
| **150** | 1.58 | 1.67 | 2.04 | 5.03 |
| **185** | 1.58 | 1.79 | 2.30 | 5.88 |
| **220** | 1.55 | 1.64 | 2.07 | 2.68 |
| **260** | 1.56 | 1.63 | 2.06 | - |
| **330** | 1.54 | 1.63 | 2.08 | - |
| **360** | 1.61 | 1.71 | 2.06 | 4.69 |
| **400** | 1.52 | 1.66 | 2.13 | - |

1. Biomass specific rates (raw data)

Table S2 Raw data of steady-state and average feast-famine biomass specific rates with their associated standard deviations. The third column shows data from the test experiments with the 13 s feeding.

|  | **Steady-state** | | **Feast-famine**  **(cycle average)**  **with 20 s feeding** | | **Feast-famine (cycle average)**  **with 13 s feeding** |
| --- | --- | --- | --- | --- | --- |
| **Working volume** (L) | 0.947 ± 0.001 | | 0.947 ± 0.001 | | 0.883 ± 0.001 |
| **Biomass** **concentration**  (g L^-1^) | 9.71 ± 0.63 | | 6.57 ± 0.15 | | 6.57 ± 0.23 |
| **Biomass growth** **μ**  (g g_CDW_^-1^ h^-1^) | 0.044 ± 0.004 | | 0.048 ± 0.009 | | 0.05 ± 0.003 |
| **q_Glucose_** (mmol_glc_ g_CDW_^-1^ h^-1^) | -0.70 ± 0.05 | | -1.07 ± 0.03 | | -1.16 ± 0.05 |
| **q_O2_** (mmol_O2_ g_CDW_^-1^ h^-1^) | -2.16 ± 0.16 | | -4.22 ± 0.19 | | -4.17 ± 0.08 |
| **q_CO2_** (mmol_CO2_ g_CDW_^-1^ h^-1^) | 2.21 ± 0.15 | | 4.35 ± 0.12 | | 4.50 ± 0.08 |
| **Respiratory Quotient** | 1.02 ± 0.10 | | 1.03 ± 0.04 | | 1.08 ± 0.01 |
|  | **Raw** | **Reconciled** | **Raw** | **Reconciled** |  |
| **q_acetate_** (mmol_ace_ g_CDW_^-1^ h^-1^) | 0.005 ± 0.0004 | 0.005 ± 0.0002 | 0.016 ± 0.003 | 0.016 ± 0.003 | N.A.* |
| **q_ethanol_** (mmol_eth_ g_CDW_^-1^ h^-1^) | 0.039 ± 0.004 | 0.041 ± 0.003 | 0.028 ± 0.012 | 0.029 ± 0.012 | N.A. |
| **q_formate_** (mmol_form_ g_CDW_^-1^ h^-1^) | 0.002 ± 0.0001 | 0.002 ± 0.0001 | 0.012 ± 0.003 | 0.013 ± 0.003 | N.A. |
| **q_lactate_** (mmol_lac_ g_CDW_^-1^ h^-1^) | 0.021 ± 0.002 | 0.022 ± 0.001 | 0.011 ± 0.002 | 0.013 ± 0.002 | N.A. |
| **Biomass Yield** (g_CDW_ g_glc_^-1^) | 0.32 ± 0.04 | | 0.22 ± 0.04 | | 0.22 ± 0.01 |
| **Oxygen Yield**  (mmol_O2_ mmol_glc_^-1^) | 3.09 ± 0.31 | | 3.94 ± 0.15 | | 3.59 ± 0.16 |
| **Carbon recovery** (%) | 101.8 | | 101.6 | | N.A. |
| **Electron recovery** (%) | 112.3 | | 108.7 | | N.A. |

*N.A.: Not available

1. Elemental analysis and Microscopy

For the elemental analysis performed, carbon (C), hydrogen (H) and nitrogen (N) were quantified with a CHN-Analyzer, phosphorus (P) was quantified with UV/VIS and sulphur (S) with ion chromatography. The measurements were performed by Mikroanalytisches Laboratorium Kolbe, Oberhausen, Germany. The oxygen content was calculated, assuming that biomass was composed only by C, H, N, P, S, O and 3% metals.

Table S3 Elemental composition of dried biomass in steady-state and feast-famine regime. The values represent grams of elements per 100 grams of dried biomass. Standard errors were derived by duplicate samples of each regime.

| **Composition** | **C (%)** | **H (%)** | **N (%)** | **P (%)** | **S (%)** | **O (%)** |
| --- | --- | --- | --- | --- | --- | --- |
| **Steady-state** | 48.88 ± 0.01 | 7.56 ± 0.01 | 15.23 ± 0.01 | 5.03 ± 0.02 | 1.33 ± 0.01 | 18.99 ± 0.03 |
| **Feast-famine** | 48.69 ± 0.01 | 7.48 ± 0.01 | 15.53 ± 0.01 | 5.17 ± 0.02 | 1.25 ± 0.01 | 18.89 ± 0.03 |
| **Change (%)** | -0.39 ± 0.03 | -1.06 ± 0.19 | +1.97 ± 0.09 | +2.78 ± 0.56 | -6.02 ± 1.07 | -0.53 ± 0.22 |


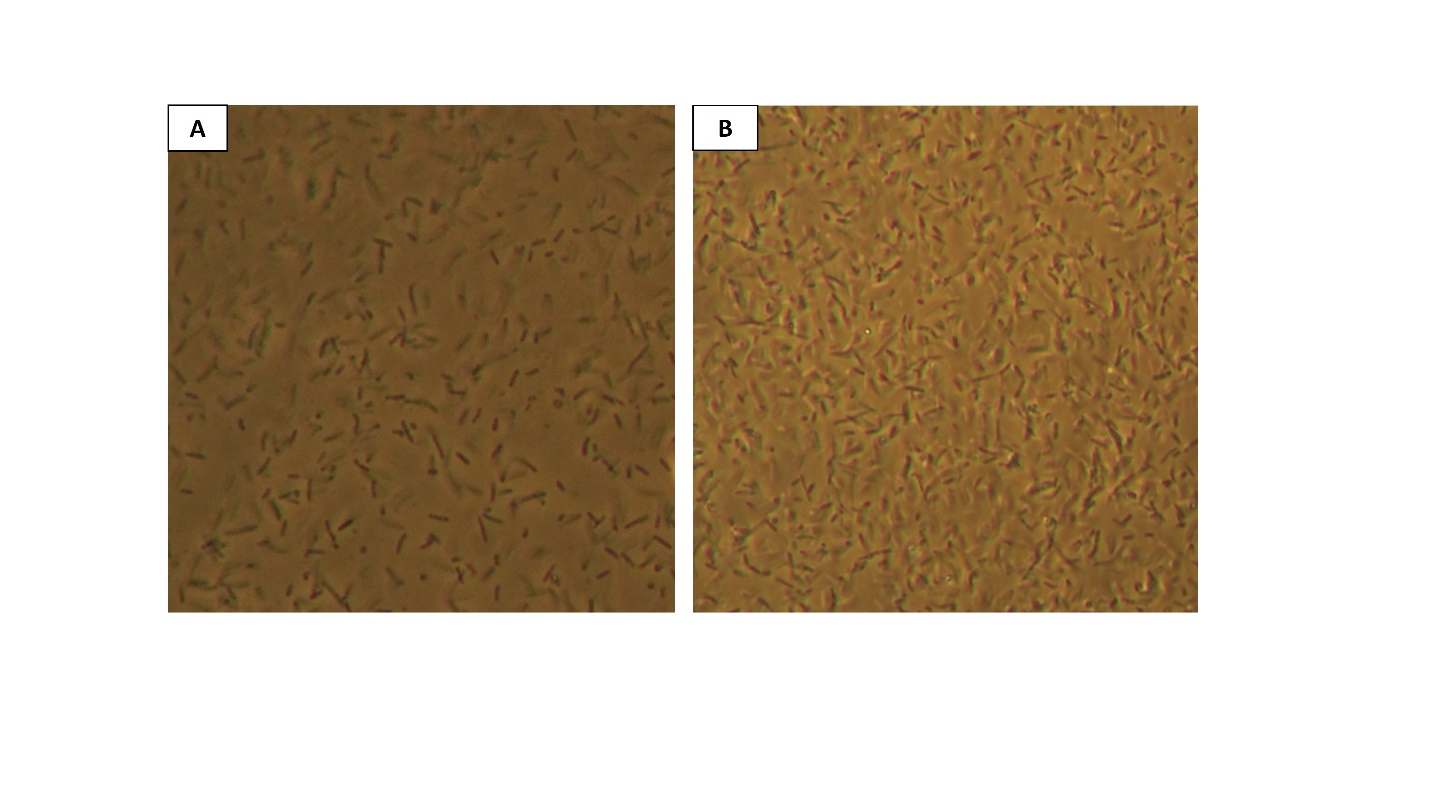


Figure S2 Optical microscope (Zeiss Axiostar Plus) images from *E.coli* cells during (A) reference steady-state growth and (B) feast-famine growth. 1000x zoom was used and the dilution of the samples was not the same.

1. Reaction list used in FBA

| **Flux Abbreviations** | **Enzymes** | **Reactions** |
| --- | --- | --- |
| 1. PTS | Phosphotransferase system enzymes | Glucose + PEP 🡪 G6P + PEP_out_ |
| 1. G6PDH | Glucose-6-phosphate dehydrogenase | G6P 🡪 6PG |
| 1. PGI | Glucose-6-phosphate isomerase | G6P ↔ F6P |
| 1. PFK | Phosphofructokinase | F6P ↔ FBP |
| 1. FBA | Fructose-biphosphate aldolase | FBP ↔ DHAP + GAP |
| 1. TPI | Triose-phosphate isomerase | DHAP ↔ GAP |
| 1. GAPD/PGK | Glyceraldehyde-3-phosphate/Phosphoglycerate kinase | GAP ↔ 3PG |
| 1. PGM | Phosphoglycerate mutase | 3PG ↔ 2PG |
| 1. ENO | Enolase | 2PG ↔ PEP |
| 1. PYK | Pyruvate kinase | PEP 🡪 PEP_out_ |
| 1. GND/RPE | 6-phosphogluconate dehydrogenase/Ribulose-phosphate 3-epimerase | 6PG ↔ Xyl5P |
| 1. GND/RPI | 6-phosphogluconate dehydrogenase/Ribose-5-phosphate isomerase | 6PG ↔ Rib5P |
| 1. TKT1 | Transketolase 1 | Xyl5P + Rib5P ↔ GAP + S7P |
| 1. TKT2 | Transketolase 2 | Xyl5P + E4P ↔ F6P + GAP |
| 1. TALA | Transaldolase A | GAP + S7P ↔ F6P + E4P |

Metabolites with the subscript “out” are products outside of the balancing space. We assume that pyruvate is the product of the PYK reaction.

1. Flux balance analysis – Derived fluxes

Table S4 FBA estimated fluxes in μmol_substrate_/g_CDW_/s.

|  | **Fluxes (Glycolysis)** | | | | | | | | | |
| --- | --- | --- | --- | --- | --- | --- | --- | --- | --- | --- |
|  | PTS | G6PDH | PGI | PFK | FBA | TPI | GAPD/PGK | PGM | ENO | PYK |
| **Time (s)** |  |  |  |  |  |  |  |  |  |  |
| 0 | 0.005 | 0.000 | 0.017 | 0.022 | 0.034 | 0.042 | 0.076 | 0.069 | 0.067 | 0.050 |
| 2 | 4.676 | 2.681 | 2.920 | 4.905 | 4.932 | 4.990 | 10.810 | 10.243 | 10.192 | 5.149 |
| 15 | 4.404 | 1.885 | 2.123 | 3.448 | 4.019 | 4.211 | 8.908 | 8.975 | 8.981 | 4.676 |
| 18 | 1.042 | 0.382 | 0.620 | 0.854 | 0.800 | 0.784 | 1.706 | 1.742 | 1.745 | 0.717 |
| 110 | 0.022 | 0.000 | 0.018 | 0.021 | 0.008 | 0.000 | 0.000 | 0.003 | 0.005 | 0.000 |
| 400 | 0.005 | 0.000 | 0.017 | 0.022 | 0.034 | 0.042 | 0.076 | 0.069 | 0.067 | 0.050 |
|  |  |  |  |  |  |  |  |  |  |  |
|  | **Fluxes (Pentose phosphate pathway)** | | | | | | | | | |
|  | GND/RPE | GND/RPI | TKT1 | TKT2 | TALA |  |  |  |  |  |
| 0 | 0.001 | 0.0002 | 0.0002 | 0.001 | 0.001 |  |  |  |  |  |
| 2 | 1.823 | 0.874 | 0.890 | 0.924 | 0.924 |  |  |  |  |  |
| 15 | 1.302 | 0.658 | 0.671 | 0.674 | 0.673 |  |  |  |  |  |
| 18 | 0.248 | 0.122 | 0.123 | 0.121 | 0.121 |  |  |  |  |  |
| 110 | 0.006 | 0.000 | 0.000 | 0.010 | 0.000 |  |  |  |  |  |
| 400 | 0.014 | 0.0002 | 0.0002 | 0.001 | 0.001 |  |  |  |  |  |

|  |  |  |  |  |  |  |  |  |  |  |
| --- | --- | --- | --- | --- | --- | --- | --- | --- | --- | --- |

1. Amino acids


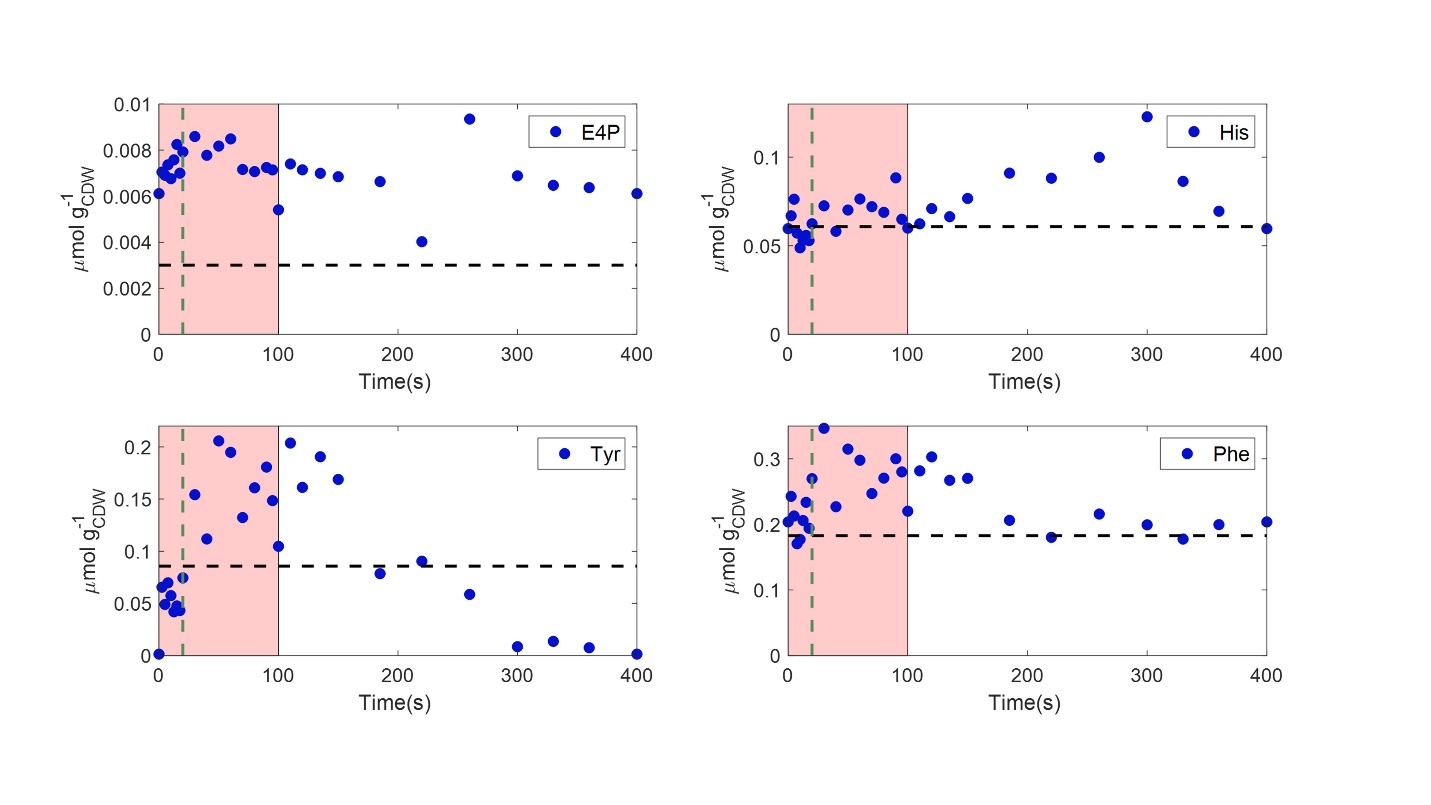


Figure S3 Intracellular concentrations (μmol/g_CDW_) of amino acids (histidine, tyrosine and phenylalanine) with E4P as a precursor, over a feast-famine cycle (s). Black horizontal dashed lines represent the average steady-state levels. Green vertical dashed lines show the end of the feeding (20 s). The pink area represents the substrate feast phase.


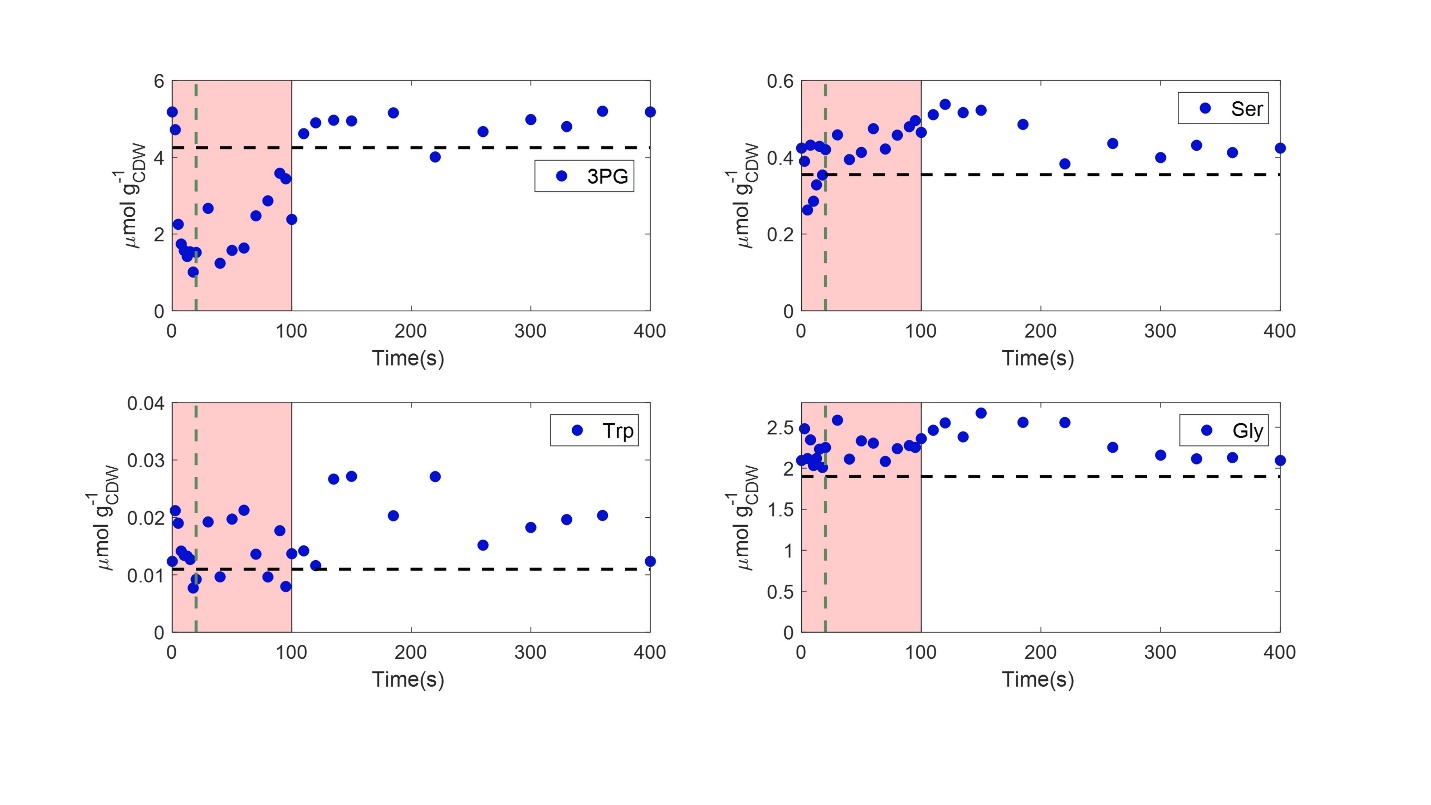


Figure S4 Intracellular concentrations (μmol/g_CDW_) of amino acids (serine, tryptophan and glycine) with 3PG as a precursor, over a feast-famine cycle (s). Black horizontal dashed lines represent the average steady-state levels. Green vertical dashed lines show the end of the feeding (20 s). The pink area represents the substrate feast phase.


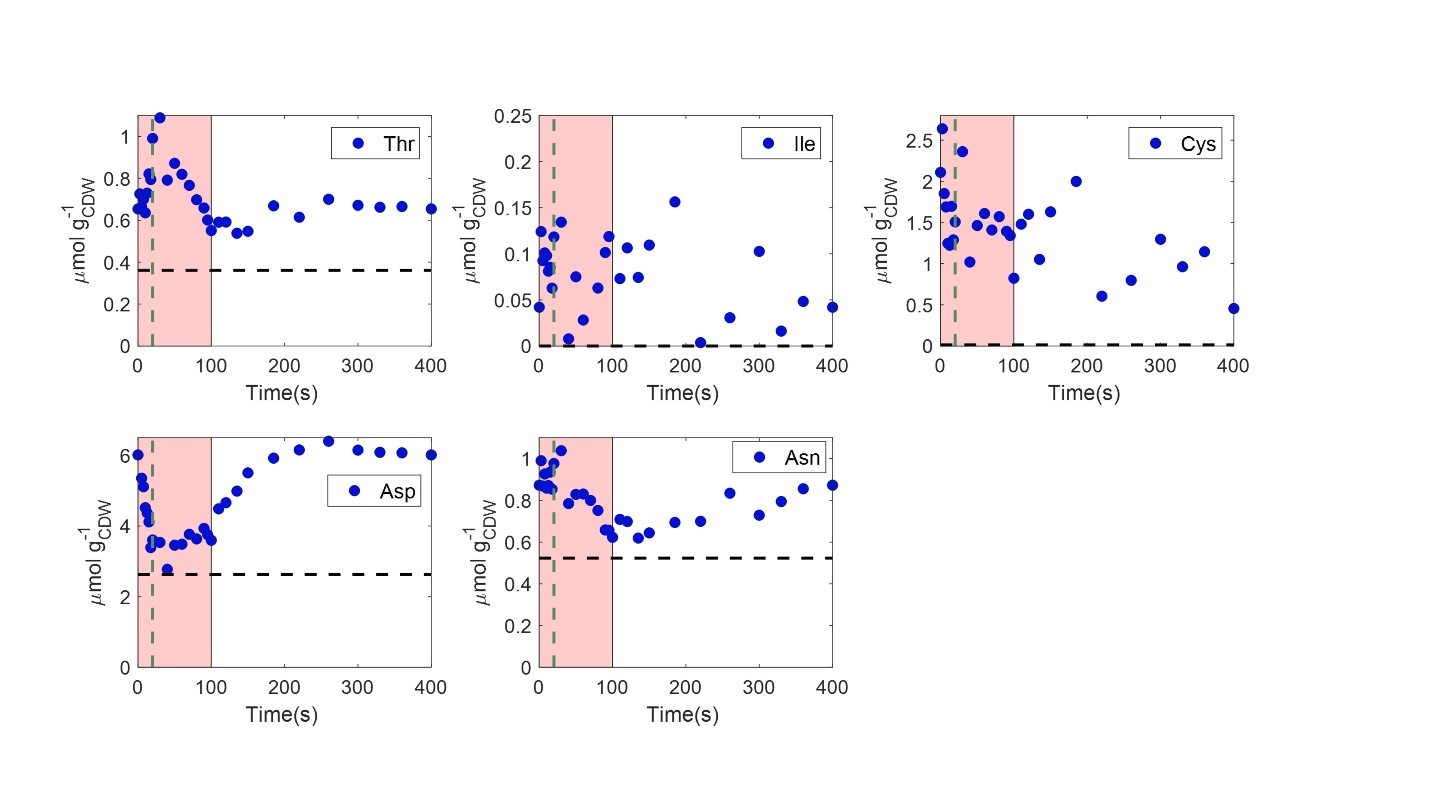


Figure S5 Intracellular concentrations (μmol/g_CDW_) of amino acids (threonine, isoleucine, cysteine, aspartate and asparagine) with oxaloacetate as a precursor, over a feast-famine cycle (s). Black horizontal dashed lines represent the average steady-state levels. Green vertical dashed lines show the end of the feeding (20 s). The pink area represents the substrate feast phase.


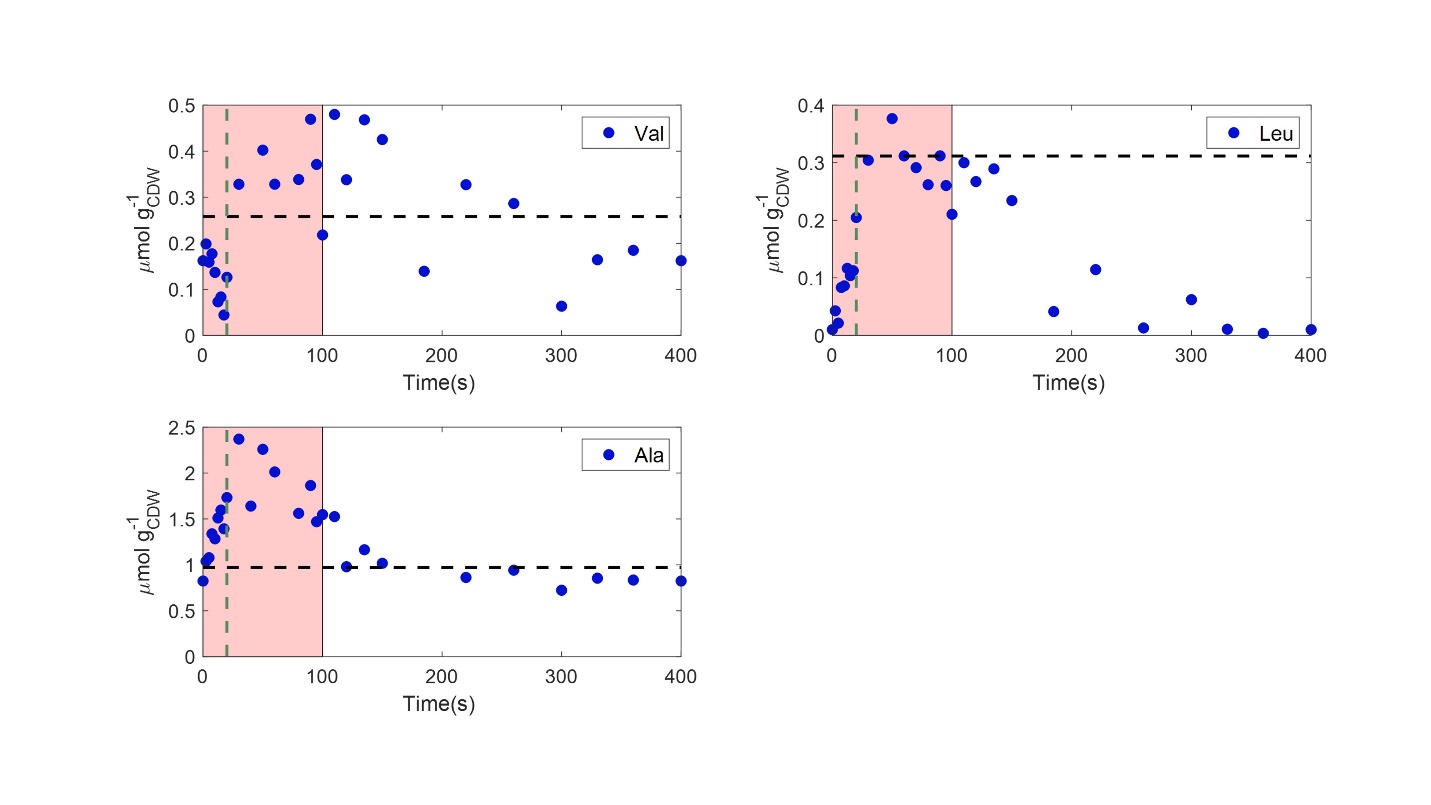


Figure S6 Intracellular concentrations (μmol/g_CDW_) of amino acids (valine, leucine and alanine) with pyruvate as a precursor, over a feast-famine cycle (s). Black horizontal dashed lines represent the average steady-state levels. Green vertical dashed lines show the end of the feeding (20 s). The pink area represents the substrate feast phase.


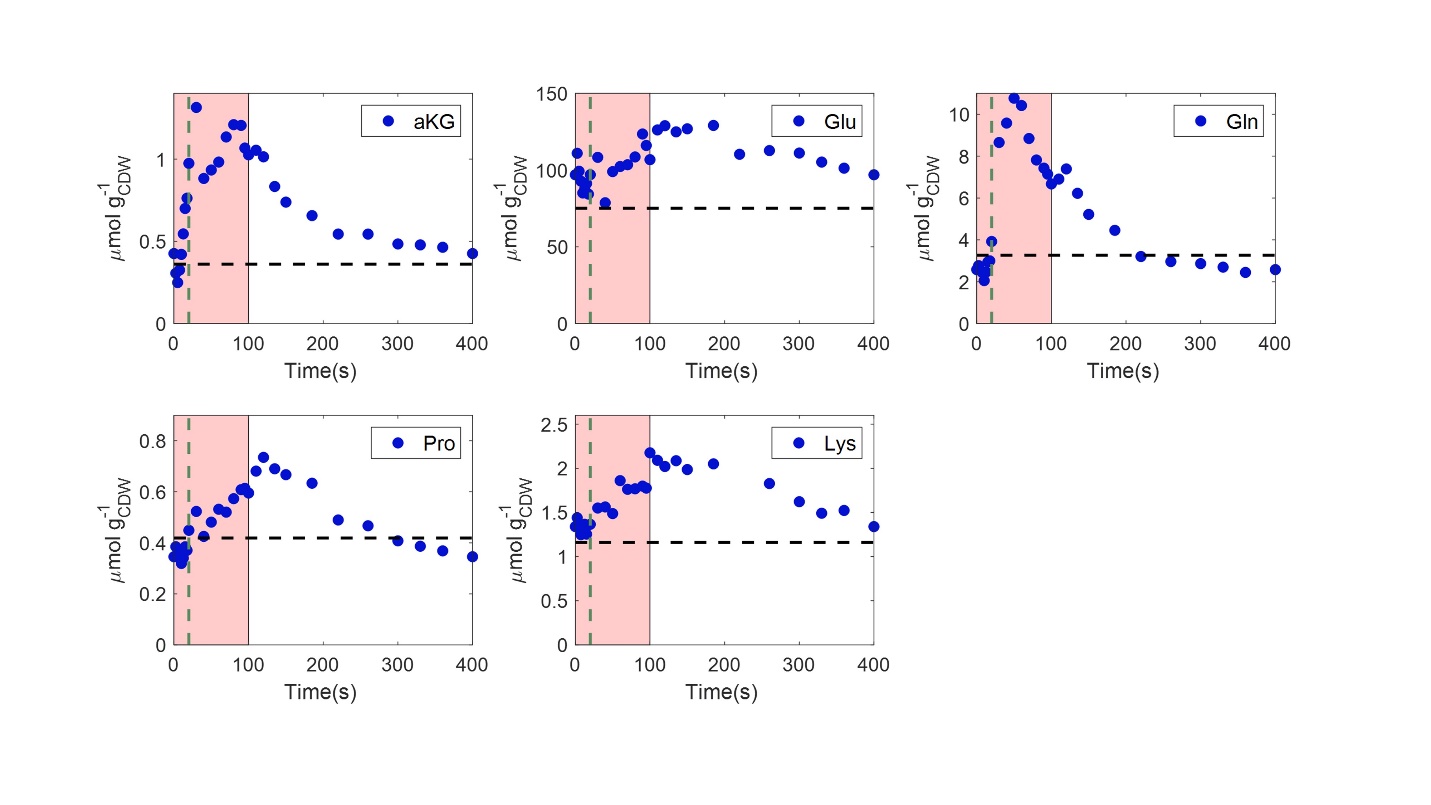


Figure S7 Intracellular concentrations (μmol/g_CDW_) of amino acids (glutamate, glutamine, proline and lysine) with aKG as a precursor, over a feast-famine cycle (s). Black horizontal dashed lines represent the average steady-state levels. Green vertical dashed lines show the end of the feeding (20 s). The pink area represents the substrate feast phase.

1. Total metabolome

Table S5 List of metabolites quantified in this study.

| **Central Carbon** | **Amino acids** | **Nucleotides** | **Rest** |
| --- | --- | --- | --- |
| Fumarate (Fum) | Alanine (Ala) | Adenosine diphosphate (ADP) | Trehalose (Tre) |
| Malate (Mal) | Glycine (Gly) | Adenosine triphosphate (ATP) | Trehalose-6-phosphate (T6P) |
| alpha-ketoglutarate (aKG) | Valine (Val) | Uridine triphosphate (UTP) | Mannose-6-phosphate (M6P) |
| Glyceraldehydephosphate (GAP) | Leucine (Leu) | Uridine diphosphate (UDP) | Uridine diphosphate glucose (UDP-glucose) |
| Citrate (Cit) | Isoleucine (Ile) | Guanosine diphosphate (GDP) | Mannitol-1-phosphate (M1P) |
| Isocitrate (iCit) | Proline (Pro) | Guanosine triphosphate (GTP) | Glucose-1-phosphate (G1P) |
| 2-phosphoglycerate (2PG) | Serine (Ser) |  |  |
| 3-phosphoglycerate (3PG) | Threonine (Thr) |  |  |
| Dihydroacetonephosphate (DHAP) | Methionine (Meth) |  |  |
| Erythrose-4-phosphate (E4P) | Aspartate (Asp) |  |  |
| Ribose-5-phosphate (Rib5P) | Phenylalanine (Phe) |  |  |
| Xylose-5-phosphate (Xyl5P) | Glutamate (Glu) |  |  |
| Fructose-6-phosphate (F6P) | Lysine (Lys) |  |  |
| Glucose-6-phosphate (G6P) | Asparagine (Asn) |  |  |
| Sedoheptulose-7-phosphate (S7P) | Glutamine (Gln) |  |  |
| Fructobiphosphate (FBP) | Tyrosine (Tyr) |  |  |
| Phosphoenolpyruvate (PEP) | Histidine (His) |  |  |
| Succinate (Suc) | Cysteine (Cys) |  |  |
| 6-phosphogluconate (6PG) | Tryptophan (Trp) |  |  |

**References**

1. Philichi TL, Stenstrom MK: **Effects of Dissolved-Oxygen Probe Lag on Oxygen-Transfer Parameter-Estimation.** *Journal Water Pollution Control Federation* 1989, **61:**83-86.

2. Vanrolleghem PA, Spanjers H: **A hybrid respirometric method for more reliable assessment of activated sludge model parameter.** *Water Science and Technology* 1998, **37:**237-246.

3. Smith CA, Corripio AB: *Principles and practice of automatic process control.* Hoboken, NJ: Wiley; 2006.

4. Vieth E: **Fitting piecewise linear regression functions to biological responses.** *J Appl Physiol (1985)* 1989, **67:**390-396.

5. Schumacher R, Wahl SA: **Effective Estimation of Dynamic Metabolic Fluxes Using (13)C Labeling and Piecewise Affine Approximation: From Theory to Practical Applicability.** *Metabolites* 2015, **5:**697-719.

6. Wang X, Conway W, Burns R, McCann N, Maeder M: **Comprehensive study of the hydration and dehydration reactions of carbon dioxide in aqueous solution.** *J Phys Chem A* 2010, **114:**1734-1740.

7. Sperandio M, Paul E: **Determination of carbon dioxide evolution rate using on-line gas analysis during dynamic biodegradation experiments.** *Biotechnol Bioeng* 1997, **53:**243-252.

8. Minkevich IG, Neubert M: **Influence of Carbon-Dioxide Solubility on the Accuracy of Measurements of Carbon-Dioxide Production-Rate by Gas Balance Technique.** *Acta Biotechnologica* 1985, **5:**137-143.

9. Dickson AG, Millero FJ: **A Comparison of the Equilibrium-Constants for the Dissociation of Carbonic-Acid in Seawater Media.** *Deep-Sea Research Part a-Oceanographic Research Papers* 1987, **34:**1733-1743.
